# Could Nature Contribute to the Management of ADHD in Children? A Systematic Review

**DOI:** 10.3390/ijerph21060736

**Published:** 2024-06-05

**Authors:** Maddison Hood, Oliver Baumann

**Affiliations:** School of Psychology, Bond University, Robina, QLD 4226, Australia; maddison.hood@student.bond.edu.au

**Keywords:** ADHD, nature, children, adolescents, attention restoration

## Abstract

Attention-deficit/hyperactivity disorder (ADHD) is a common neurodevelopmental disorder that is typically managed with pharmacological and psychotherapeutic interventions. In the general population, exposure to nature has been found to have robust beneficial effects on cognitive performance, including attention. With inattention being a factor of the symptomatology of individuals with ADHD, this provides a rationale to investigate the potential benefits of exposure to nature for this population. Four electronic databases (PubMED, PsycINFO, Embase, and Web of Science) were searched for empirical studies investigating the effects of nature on ADHD prevalence and/or symptom severity in populations of school-aged children. Key characteristics, methodologies, and outcomes of included studies were extracted and evaluated. Out of the 458 studies identified, 7 met the inclusion criteria. Despite the large heterogeneity in methodological approaches, the included articles consistently reported that exposure to nature is associated with reduced ADHD diagnoses and symptom severity. Furthermore, when several covariates, such as age, gender, annual household income, parental income, and education level, as well as several pre-natal factors, were controlled for, the relationship between nature and ADHD remained significant. The reviewed literature provides strong support for the benefits of exposure to nature on ADHD in school-aged children.

## 1. Introduction

Attention-deficit/hyperactivity disorder (ADHD) is a common neurodevelopmental disorder often diagnosed in childhood. It is characterized according to the *Diagnostic and Statistical Manual for Psychiatric Disorders, Fifth Edition* [1] (DSM-5; American Psychiatric Association) by a persistent pattern of inattention and/or hyperactivity—impulsivity that interferes with functioning and is persistent across multiple settings. Systematic reviews and meta-analyses have suggested that the global prevalence of ADHD is between 2 and 7% [2], with 5% of individuals 18 years of age or younger meeting the DSM-5 or International Classification of Disease (ICD-10; World Health Organization; [3]) criteria [4].

While the causes of ADHD are still being investigated, it is understood that ADHD has clear neurobiological underpinnings and that the heritability of ADHD is substantial, with first-degree biological relatives of individuals with ADHD posing a significant risk factor for the development of the disorder [5,6,7]. Additionally, several factors such as very low birth weight, maternal smoking during pregnancy, alcohol exposure in utero, and certain childhood temperaments have been associated with ADHD (DSM-5; [1]).

### 1.1. Current ADHD Treatment Options

Typically, the management of ADHD consists of pharmacological and non-pharmacological interventions [8]. While there is support for the efficacy of pharmacological approaches [9], there are also disadvantages that come with this treatment approach for some patients, including negative side effects, little or no symptom reduction, or medication resistance [10]. There is also scarce evidence surrounding the use of medications to treat ADHD in preschool-aged children; therefore, behavioral interventions are the first point of call for this age group [8,11]. Non-pharmacological approaches such as dietary adjustments, behavioral interventions (e.g., parent training), and neurocognitive therapies have been found to have some efficacy in the treatment of ADHD in children and adolescents [12,13,14,15]. However, evidence for the efficacy of these approaches is difficult to determine due to the broad scope of interventions and outcome measures used. The benefits of exposure to green spaces and nature seen in non-clinical populations has inspired an emergence of thought around how the physical environment could contribute to the management of ADHD in children and adolescents [16]. 

### 1.2. Benefits of Exposure to Nature

Exposure to nature has been found to have a positive impact on human health, wellbeing, and cognitive functioning [17,18,19,20,21]. More specifically, green spaces have also been shown to have benefits for restoring attention in adult and child non-clinical populations [22,23]. The Attention Restoration Theory (ART [24]) postulates that being immersed in nature actually restores cognitive functioning and increases attentional capacity. With one of the primary symptoms of ADHD being inattention, this theory is most relevant in relation to ADHD. This theory is based on the premise that there are two types of attention, direct/voluntary attention and involuntary attention [25]. According to ART, natural environments assist in the recovery from the fatigue that occurs when using direct attention because they primarily draw on involuntary attention [24]. ART also suggests that the use of involuntary attention allows a restoration of fatigued direct attention [24]. While there is evidence to suggest that the exposure to nature supports attention restoration in adults (for example [26] Berman et al., 2008), few studies have explored this concept in children [23]. It is important to note that ART primarily focuses on the exposure to nature, and it does not explicitly specify whether the type of activity performed in nature matters.

### 1.3. The Current Review

Despite there being evidence to suggest that there is a link between the exposure to nature and the restoration of attention [22,23,26], there has not yet been a systematic review of the literature investigating how this relationship might benefit individuals affected by ADHD. This review aims to address this by compiling, clarifying, and summarizing the existing evidence for the use of nature exposure in children and adolescents with ADHD and its effects on ADHD symptoms.

## 2. Materials and Methods

This systematic review followed the general principles published by the Preferred Reporting Items for Systematic reviews and Meta-Analyses statement guidelines (PRISMA; [27,28]) and sought to answer the following question: does exposure to nature have a beneficial effect on children with ADHD?

### 2.1. Literature Search

The following electronic databases were searched: PubMED, PsycINFO, Embase, and Web of Science. The following search terms were used, with appropriate Boolean operators and MeSH terms identified for each database: (Child*, Adolescent*, Student*), (ADHD, “attention deficit/hyperactivity disorder”, “attention deficit hyperactivity disorder”), (greenspace, “green space”, greenery, greenness, biophilia, “biophilic design”, plant*, natural, nature). The search was limited to humans and articles in the English language. The last search was conducted on 31 March 2021.

### 2.2. Selection Criteria

#### 2.2.1. Study Design

Studies of a quantitative nature were considered for this review, while studies with qualitative designs were excluded. The decision to exclude qualitative studies was made because the focus of this review was to analyze the empirical evidence of the effects that nature has on the symptoms of ADHD in children rather than understanding the perspectives and experience of children with ADHD.

#### 2.2.2. Population

The population of participants included preschool- and school-aged children aged below 18 years with a formal diagnosis of ADHD. Each participant underwent a diagnosis by a qualified healthcare professional using recognized and standardized assessment procedures.

#### 2.2.3. Exposure

Studies were included if they involved some type of exposure to nature (forests, parks, green spaces, gardens), and this was measured empirically either by objective measures (such as remote sensing measurements) or subjective measures (such as standardized questionnaires).

#### 2.2.4. Outcome

Studies were included if they measured ADHD at a population level (such as diagnoses of prevalence) or at an individual level (measuring symptom severity).

### 2.3. Data Collection and Evaluation of Evidence

A meta-analysis was not possible due to the heterogeneity in methodological approaches and the diverse nature of the outcome measures used; therefore, descriptive synthesis was used. Relevant study characteristics, methodologies, and outcomes of the studies included in this review were identified, extracted manually, and compiled in a data extraction table (Table 1). Information that was extracted included the following: author(s), year of publication, country, study design, study population, sample size, exposure assessment, outcome assessment, confounding factors, and main results. This information was used to create a summary of the key characteristics, methodologies, and outcomes of the included studies.

## 3. Results

### 3.1. Study Selection

**Figure 1:**
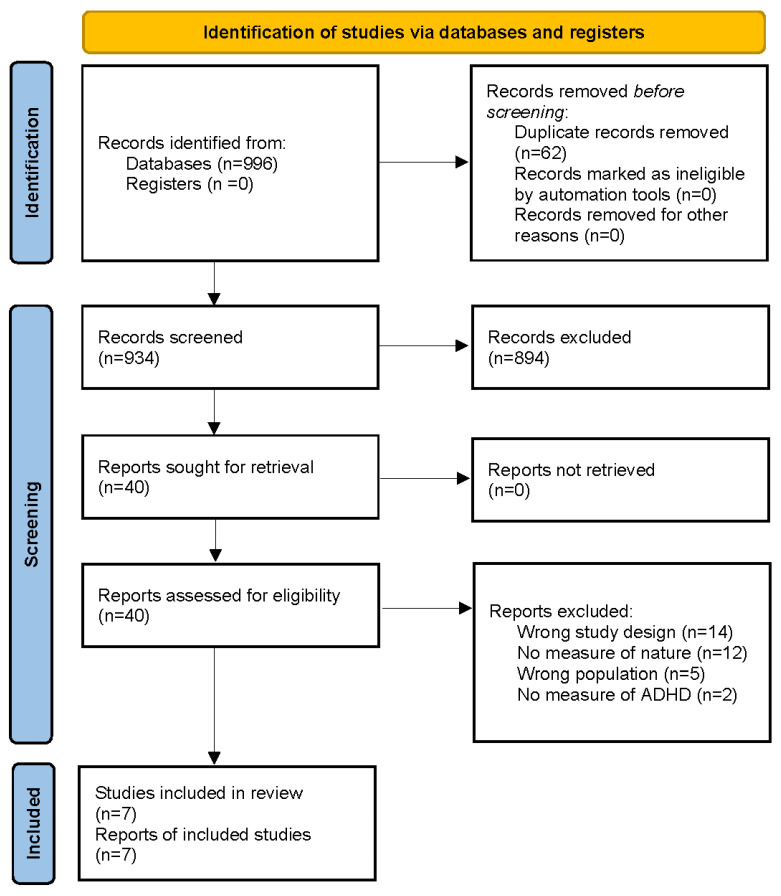
PRISMA flow chart.

Searching databases resulted in 996 studies (Figure 1). After the removal of 62 duplicates, the titles and abstracts of 934 papers were screened independently by two reviewers for eligibility. The information included in the titles and abstracts was considered based on the selection criteria to determine eligibility. If there was insufficient information in the title and abstract to determine clear eligibility, the full text was also screened. After screening based on titles and abstracts, 894 papers were deemed ineligible based on selection criteria. The full text of 40 articles was read to determine their eligibility, of which 7 articles were deemed eligible for inclusion. Of the articles that were read fully, 33 were excluded: 14 for ineligible study designs, 12 for the absence of a measure of nature, 5 for containing ineligible populations, and 2 for ineligible outcome measures. There was no disagreement among the two reviewers regarding study selection.

### 3.2. Description of Articles

As presented in Table 1, the seven selected studies were conducted in different countries and varied considerably in study design and sample size. The majority of studies employed observational designs (five out of seven), while two articles utilized experimental or quasi-experimental designs (please refer to Table 1). The majority of the included articles relied on parents’ self-reports with regard to ADHD diagnosis and symptoms. Four studies relied on parents to report that their child were formally diagnosed with ADHD by a physician, psychologist, pediatrician, or psychiatrist [29,30,31,32], and one study relied on parents’ ratings of the frequency of symptoms to categorize children as having ADHD or not [33]. More details on the parent-reported methods used in each study to determine ADHD classification can be found in Table 1. Two studies identified children with a diagnosis of ADHD from hospital records; pharmacy records containing two or more prescriptions for methylphenidate, hydrochloride, atomoxetine, or dexamfetamine sulfate [34]; and healthcare claim data [35]. Only three articles conducted analyses to assess if there were differences in effects between the different subtypes of ADHD. Two of these did not find significant statistical differences [31,33], while the study by Faber Taylor and Kuo [30] found that, for children with hyperkinetic symptoms, only spaces that are both green and open lead to improvements in symptom severity.

### 3.3. Effects of Nature on ADHD

Despite there being differences in the design and methodology of the studies, the included articles generally reported favorable effects of nature on ADHD symptoms. Studies that categorized nature based on satellite imaging reported potentially protective and/or curative factors of higher vegetation density on ADHD [33,34,35], consistently reporting that areas with a higher density of green vegetation were associated with lower instances of ADHD symptoms. Markevych and colleagues [35] demonstrated a negative correlation between ADHD and green vegetation density, while Donovan and colleagues [34] found that children who resided in rural areas were less likely to develop ADHD. Two articles that classified natural settings as those with “big trees and grass” and “a lot of open grass” reported that children who spent time in these natural settings, as reported by their parents, had less severe symptoms of ADHD and better concentration capabilities than those who spent their time in indoor or built settings, according to their parents [30,31]. Two studies that classified natural settings as “an urban park” and “an open spot in a nearby wooded area” found that children concentrated better when they were exposed to these natural settings compared to built settings and that children rated natural settings as more restorative, fun, and relaxing than the built settings [29,32].

### 3.4. Moderating and Confounding Variables

The included articles measured and analyzed fourteen potential moderating and/or confounding variables for their impact on the effect of nature on ADHD. Five articles included sex/gender in their analysis and all five found that there was no difference in the effect of nature on ADHD symptoms between males and females [30,31,33,34,35]. Four articles investigated the age of children as a potential moderating variable and found that there was no impact of age on the effects of nature on ADHD [31,33,34,35]. Annual household income was investigated by three articles and was found to have no impact on the effect of nature on ADHD [31,33,34]. In one study, household income was found to be negatively correlated with the severity of ADHD symptoms (F_(1, 386)_ = 4.75, *p* < 0.05), suggesting that children in wealthier households had milder symptoms [31]. Parental education level was also measured and reported on by two studies that found it had no impact on the relationship between nature and ADHD symptoms [30,33]. Yang and colleagues [33] found that the effects of nature on ADHD were not dependent on maternal smoking and alcohol consumption during pregnancy, preterm birth, birthweight, breastfeeding, or the number of siblings. Two studies did not report any investigation of confounding or moderating variables [29,32].

## 4. Discussion

ADHD is a neurodevelopmental disorder characterized by symptoms of inattention, hyperactivity, and impulsivity. Neurobiologically, ADHD has been associated with abnormalities in brain structure and function, particularly in regions involved in executive function and attention regulation, such as the prefrontal cortex and the basal ganglia [36]. Dopaminergic and noradrenergic systems, which are crucial for attention and behavioral control, are also often dysregulated in individuals with ADHD [37]. Nature may influence these brain systems by providing a serene environment that reduces stress and cortisol levels, having the ability to positively affect the prefrontal cortex, which is involved in executive functions [38,39].

The reviewed literature suggests an overall favorable effect of exposure to nature on ADHD. There are, however, a variety of ways in which nature has been defined and measured in the literature, and it is useful to consider the strengths and limitations of these different approaches to help guide and refine the future research in this area. Three studies included in this review used large-scale satellite imaging to identify and classify nature based on the ‘greenness’ in the images [30,33,35]. On the one hand, this approach has the advantage that it is objective and replicable; on the other hand, however, this analysis does not allow for a more fine-grained classification of different nature settings (e.g., large trees vs. small trees) and it ignores other aspects of nature, such as water, that are not green vegetation but may still have a beneficial effect for children with ADHD. In contrast to the satellite imaging approach, several other studies [29,31,32,34] employed a more descriptive and idiosyncratic approach to classify nature (e.g., “urban park”), which has the advantage of providing a finer-grained classification but somewhat lacks objectivity and comparability across different studies.

Furthermore, once nature has been classified, there is the question of how to quantify the exposure to it. At one end of the spectrum, there are satellite imaging studies [30,33,35], which defined exposure to nature according to the geographical location of the participants’ home address. On the other end of the spectrum are studies that employed self-reports by either the researcher(s) [31,34] or parents [29,32] to quantify the exposure to nature. Weeland and colleagues [40] assessed the impacts of nature on primary school-aged children’s’ ability to self-regulate their emotions and found stronger positive effects when exposure was measured via parent reports than via an objective index, such as a normalized difference vegetation index. This could indicate that qualitative aspects of experiencing nature are more predictive than the quantity of vegetation. However, while self-reports provide a richer account of exposure to nature, they somewhat lack objectivity and comparability across different studies. Furthermore, in future investigations examining the impact of nature on ADHD, it is crucial to explore specific natural elements, such as bird songs and water, to thoroughly assess their therapeutic potential.

While the included studies demonstrated a clear positive effect of exposure to nature on ADHD symptoms in children, it is important to note that the reviewed studies were mostly observational. Moreover, they may not have yet captured the minimum level of exposure required to achieve an effect. A survey of the general adult population has shown that there is a dose-dependent effect for the duration spent in nature on the prevalence of depression [41]. Many families may have difficulty accessing nature, and schools as well as other communities have typically limited resources. Therefore, it is important to establish a minimum intensity and duration of exposure to nature to provide an understanding with regard to the effects on ADHD symptomology that can be achieved, even with minimal interventions. Additionally, it would be useful to measure the longevity of these effects by using a longitudinal design, with follow-up measures. This would help inform real-world applications with regard to how often this intervention may need to be re-administered, if at all. It is also important to acknowledge that the reviewed studies do not allow for investigating how much the effects are caused by activities in nature or by nature alone, which is a topic that should be addressed by future research.

It Is also important to note that ADHD is a heterogeneous disorder with diverse expressions of symptom domains. Only three of the reviewed studies considered the effects of ADHD subtypes. Future studies should therefore evaluate the benefits of nature while considering the heterogeneity in the symptomology of this disorder. In this context, it is also relevant to highlight the new Research Domain Criteria (rDoC) framework of the USA-based National Institute of Mental Health, which focuses on investigating dimensional biological, physiological, and behavioral mental health factors instead of heterogenous symptom-based diagnostic categories [42]. In line with this approach, it might be fruitful to analyze effects across a spectrum of psychological functioning, irrespective of boundaries for distinct ADHD subtypes [43]. Moreover, only three out of the seven studies assessed the medication status of the participants. Among these, none analyzed medication as a moderating factor. Future studies should therefore address this gap by including medication status in their analyses to better understand its potential moderating effects.

Finally, in terms of limitations, this review was based on the analysis of only seven articles, which restricts the robustness of our conclusions. However, it remains crucial to summarize the available literature and identify areas for future research.

## 5. Conclusions

The exploration of how best to manage and treat ADHD in children is ongoing; therefore, the findings reviewed here about the benefits of nature regarding ADHD symptoms are relevant and encouraging. While further research should be conducted to support a causal effect and provide better insight into the mechanisms behind this, there is strong enough evidence to begin implementing this approach into practice for use alongside already established treatments for ADHD. One way this could be implemented is by incorporating nature into behavioral interventions by recommending an increase in exposure to nature as a method of management in itself, or by increasing the presence of nature in therapeutic spaces. In general, incorporating elements of nature in the design of built environments, such as indoor plants, water features, large windows with natural light, etc., would allow children who live in urban areas to receive the benefits of exposure to nature that they otherwise would not have. Using this in areas we know children with ADHD spend time, such as schools, homes, and therapy rooms, could be one way in which children with ADHD could be subjected to the benefits highlighted in this review. Exposure to nature should be embraced as an additional approach to managing ADHD in children and adolescents to complement and enhance the existing treatment approaches. Moreover, it should be implemented at an individual and wider community level to allow individuals with ADHD to experience the benefits of nature.

## Figures and Tables

**Table 1:** Study characteristics, methodology, and results.

Author(s), Year, Country	Study Design	*N*, Age Range, Medication Status	Nature Exposure Data Source	ADHD Measure	Outcome Measure	Significant Results
Donovan et al., 2019, New Zealand	Observational (longitudinal)	*N* = 49,923 Children <18 years old (100% on ADHD medication)	Normalized difference vegetation index (NDVI) and land use data from Landcare Research, New Zealand.	Hospital diagnosis of ADHD or pharmacy records (two or more prescriptions for methylphenidate, hydrochloride, atomoxetine, or dexamfetamine sulfate).	Presence of ADHD diagnosis.	Children who had always lived in a rural area after 2 years of age were less likely to develop ADHD (odds ratio [OR] 0.670, 95% CI 0.461–0.974), as were those with increased minimum NDVI exposure after age 2 years (standardized OR for exposure vs. first quartile: second quartile 0.841 [0.707–0.999]; third quartile 0.809 [0.548–0.805]). In early life (pre-natal to age 2 years), neither rural living nor NDVI were protective against ADHD. Neither mean nor maximum greenness was significantly protective against ADHD.
Faber Taylor and Kuo, 2009, U.S.	Single-blind experimental (within-subjects)	*N* = 16 Children 7 to 12 years old (50% on ADHD medication)	Exposure to an urban park, a downtown area, and a residential area via individually guided 20 min walks.	Parent-reported diagnosis of ADHD by physician, psychologist, or psychiatrist.	Concentration measured using Digit Span Backwards subtest from the *Weschler Intelligence Scale for Children, Fifth Edition* (WISC-V). Children’s experience was measured by asking them to rate 7 descriptions using a 3-point Likert scale.	Children with ADHD concentrated better after a walk in the park than after a downtown walk (*p* = 0.0229, Cohen’s d = 0.52) or the neighborhood walk (*p* = 0.0072, Cohen’s d = 0.77). The park setting was rated significantly higher than the other two settings on fun (*t*_13_ = 2.39, *p* = 0.02) and marginally significantly higher on *relaxing* (*t*_14_ = 1.42, *p* = 0.83).
Faber Taylor, and Kuo, 2011, U.S.	Observational (cross-sectional)	*N* = 421 Children 5–18 years old (unknown ADHD medication status)	Parent-reported questionnaire about child’s everyday play setting. Parents were asked to identify, out of 10 descriptions, which best matched their child’s play setting in the previous week, or to described this in their own words.	Parent-reported diagnosis of ADHD, ADD, or do not know.	ADHD symptom severity rated by parents using a 5-point Likert scale on 4 DMS-5 ADHD diagnostic criteria: difficulty remaining focused on unappealing tasks; difficulty in completing tasks; difficulty in listening and following directions; difficulty in resisting distractions.	An ANOVA found that there was a significant main effect of the setting on symptom severity (*F_(_*_3, 321)_ = 5.78, *p* < 0.001). Pairwise comparisons found that children playing in indoors had significantly more severe symptoms than children playing in grassy outdoor settings (*d* = 0.57, *p* < 0.001) and settings with big trees and grass (*d* = 0.25, *p* < 0.05). Children playing in built outdoor settings also had more severe symptoms than children who played grassy outdoor settings (*d* = 0.64, *p* = 0.001) and settings with big trees and grass (*d* = 0.31, *p* = 0.05). Income was also found to be significantly related to the severity of symptoms, with household income being negatively related to the severity of symptoms (*R*^2^ = 0.01, *F_(_*_1, 386)_ = 4.75, *p* < 0.05), but it was not significantly related to the play setting (*F_(_*_3, 299)_ = 0.14, *p* = 0.94).
Kuo and Faber Taylor, 2004, U.S.	Observational (cross-sectional)	*N* = 452 Parents or legal guardians of children aged 5–18 years (with formal diagnosis of ADHD; unknown ADHD medication status)	Parent-reported questionnaire about child’s everyday play setting. Parents were asked to identify, out of 10 descriptions, which best matched their child’s play setting in the previous week, or to described this in their own words.	Parent report of a formal diagnosis by a physician, psychologist, or psychiatrist.	ADHD symptom severity rated by parents using a 5-point Likert scale on 4 DMS-5 ADHD diagnostic criteria: difficulty remaining focused on unappealing tasks; difficulty in completing tasks; difficulty in listening and following directions; difficulty in resisting distractions.	Results of one sample *t*-tests indicated that green outdoor activities significantly reduced symptoms whether or not activities were conducted alone/in pairs (*t*_430_ = 16.91, *p* < 0.0001) or in larger groups (*t*_408_ = 11.65, *p* = 0.0002). A 2 × 2 (physical setting X social context) repeated-measures ANOVA indicated that the same activities reduced symptoms significantly more when conducted in green outdoor settings than when conducted in indoor settings or built outdoor settings (*F*_(1, 375)_ = 32.1, *p* < 0.001 and *F_(_*_1, 386)_ = 21.9, *p* < 0.001, respectively). Only green outdoor settings reduced symptoms, regardless of the social context. Green outdoor activities reduced symptoms significantly more than either built outdoor activities or indoor activities for the sample as a whole, regardless of the social context. Results were consistent across ages, income brackets (<USD 25,000 to >USD 75,000 per year), specifiers (i.e., ADHD and ADD), across severity, and children without comorbid conditions as well as those with a comorbid learning disorder.
Markevych et al., 2018, Germany	Observational (longitudinal)	*N* = 2044 Children born in Saxony in 2005 who were diagnosed with ADHD up to the year 2014, <18 years old (unknown ADHD medication status)	Ten-year average level of normalized difference vegetation index (NDVI) for the years 2005–2014.	ICD-10-GM F90 diagnosis by a child/adolescent psychiatrist, neuropediatric, or psychotherapist.	Presence of ADHD diagnosis.	An increase in NDVI decreased the relative risk of ADHD by a factor 0.82 [0.68–0.98]. There was a negative correlation between ADHD and NDVI (ρ = −0.31). ADHD was also correlated with the proximity to doctors, particularly child/adolescent psychiatrists and neuropediatricians (ρ = 0.48).
van den Berg, and van den Berg, 2010, Zeeland, The Netherlands	Quasi-experimental (within- subjects)	*N* = 12 Children 9–17 years (10 children on ADHD medication)	Exposure to “an open spot in a nearby wooded area” for one hour, on one day, as observed by researchers.	Parent-reported diagnosis.	*Perceived restoration:* ten questions adapted from Connectedness to Nature Scale and the Perceived Restorativeness Scale. *Mood*: self-report smiley-test. *Concentration*: ‘opposite worlds’ test from the Test of Everyday Attention for Children.	Children perceived the woods as more restorative than the town (*η*^2^ = 0.37, *p* ≤ 0.01). Children had more difficulties with concentration in the town than in the woods (*η*^2^ = 0.21, *p* = 0.07).
Yang et al., 2019, China	Observational (cross-sectional)	*N =* 59,754 Children aged 2–17 years (*N* = 2566 with ADHD symptoms; unknown ADHD medication status)	Greenness surrounding each child’s school or kindergarten was estimated using 2 satellite image-derived vegetation indexes: the normalized difference vegetation index and the soil-adjusted vegetation index within 500 m of a school or kindergarten.	Parents or guardians rated the frequency of 18 ADHD symptoms (from DSM-IV) during the preceding 6 months. Children with 6 or more symptoms of either inattention or hyperactivity-impulsivity as rated by parents/guardians were investigated.	Number of ADHD symptoms (from DSM-IV) as reported by parents/guardians.	Greater greenness levels were associated with lower odds of ADHD symptoms. Covariate-adjusted models demonstrated that a 0.1 unit increase in NDVI or soil-adjusted vegetation index within 500 m of a school or kindergarten was significantly associated with lower odds of ADHD symptoms (odds ratios, 0.87 [95% CI, 0.83–0.91] *p* < 0.01 and 0.80 [0.74–0.86] *p* < 0.001, respectively).

Notes. ADHD: attention-deficit/hyperactivity disorder; CI: confidence interval; DSM: *Diagnostic and Statistical Manual*; NDVI: normalized difference vegetation index.

## Data Availability

The original contributions presented in this study are included in this article.
